# Identification and Characterization of Influential Factors in Susceptibility to Attention Deficit Hyperactivity Disorder Among Preschool-Aged Children

**DOI:** 10.3389/fnins.2021.709374

**Published:** 2022-01-31

**Authors:** Xiangling Deng, Min Yang, Shunan Wang, Bo Zhou, Kundi Wang, Zhixin Zhang, Wenquan Niu

**Affiliations:** ^1^Graduate School, Beijing University of Chinese Medicine, Beijing, China; ^2^Department of Pediatrics, China-Japan Friendship Hospital, Beijing, China; ^3^International Medical Services, China-Japan Friendship Hospital, Beijing, China; ^4^China-Japan Friendship Hospital, Institute of Clinical Medical Sciences, Beijing, China

**Keywords:** preschool-aged children, ADHD related symptoms, risk factor, C-ASQ, nomogram model

## Abstract

Attention deficit hyperactivity disorder (ADHD) is the most common childhood-onset neurodevelopmental disorder. Currently, increasing amounts of attention have been focused on the epidemiologic profiling of ADHD in children, viewed as a continuously distributed risk dimension throughout the whole lifespan. This study aimed to identify and characterize potential influential factors susceptible to ADHD-related symptoms among preschool-aged children. A comprehensive questionnaire was self-designed for both children and their parents or guardians and was distributed to 30 kindergartens from Beijing and Hebei, collecting potential influential factors in susceptibility to ADHD. ADHD was assessed by the Conner’s Abbreviated Symptom Questionnaire (C-ASQ), and 7,938 children were analyzed. Least absolute shrinkage and selection operator (LASSO) regression and hierarchical degree of adjustment were used to control possible covariates. Five factors, namely, children’s secondhand smoking exposure, breastfeeding duration, sleep mode, maternal pregnancy smoking exposure, and parental self-rating for patience, were identified to be independently and significantly associated with ADHD susceptibility. Meanwhile, dose–response relationships were observed between breastfeeding duration, parental self-rating for patience, and ADHD-related symptoms. Finally, a nomogram model was created for predicting ADHD susceptibility based on significant and conventional attributes under each criterion.

## Introduction

Attention deficit hyperactivity disorder (ADHD) is the most common childhood-onset neurodevelopmental disorder, and it is characterized by developmentally inappropriate levels of overactivity, inattention, and impulsivity (Thapar and Cooper, 2016). Currently, increasing amounts of attention have been focused on the epidemiologic profiling of ADHD in children, viewed as a continuously distributed risk dimension throughout the whole lifespan, with pooled prevalence rates ranging from 2.73 to 4.27% in children younger than 7 years (Vasileva et al., 2020) and at 3.40% in children and adolescents (Polanczyk et al., 2015).

Longitudinal data have shown that early onset ADHD is consistently associated with emotional problems throughout childhood (Stern et al., 2020), and it exerts a significant impact on functioning during adulthood (Harstad et al., 2020). Cohort studies revealed hyperactive, impulsive, and inattention symptoms as risk factors for adult intimate partner violence (Buitelaar et al., 2020). In addition, suicidality (Garas and Balazs, 2020), automobile crashes, traffic fatalities (Aduen et al., 2018), and even conviction and incarceration (Mohr-Jensen et al., 2019) have been reported to be significantly associated with ADHD symptoms. Meanwhile, ADHD places a major burden on health and healthcare costs. National annual healthcare costs in the United States ranged from $143 billion to $266 billion a decade ago (Doshi et al., 2012). Due to these severe circumstances, the concept of ADHD has been developed and refined in response to its clinical nature and structure over the past several decades. Nonetheless, many children and youth diagnosed with ADHD still receive no treatment or insufficient treatment (Manos et al., 2017), especially in low-income and middle-income countries (Thapar and Cooper, 2016).

The exploration of risk factors has proven to be beneficial for the development of preventive and intervention strategies to curb this global burden. It is widely recognized that ADHD is a highly heritable and multifactorial disorder to which multiple inherited and non-inherited factors contribute individually and interactively (Biederman and Faraone, 2005). Environmental profiles, including prenatal and perinatal factors, environmental toxins, dietary factors, and psychosocial factors, have been reported to be associated with ADHD risk (Thapar et al., 2013). It is of great importance to construct a prediction model by incorporating multiple established influential environmental factors for childhood ADHD symptoms.

To shed some light on this issue and yield more information for further studies, we conducted a cross-sectional investigation among preschool-aged children enrolled from 20 kindergartens in Beijing and 10 kindergartens in Tangshan city to identify promising factors that can predict the risk of childhood ADHD, and to enhance the magnitude of predicting ADHD susceptibility, and further we established a nomogram model by regressing individual attributes that were significantly associated with ADHD.

## Materials and Methods

### Study Design

A population-based cross-sectional survey, in accordance with the principles of the Declaration of Helsinki, was conducted in Beijing and Tangshan city (Hebei province) between September and December 2020. This survey was reviewed and approved by the Ethics Committee of China-Japan Friendship Hospital.

### Study Subjects

Preschool-aged children (3–7 years old) who attended junior to senior kindergarten classes were surveyed in this study. Utilizing a stratified cluster random sampling strategy, 5 out of 16 districts in Beijing and 1 city (Tangshan) in Hebei, a total of 30 public kindergartens were randomly selected.

### Basic Characteristics

To collect potential influential factors for ADHD susceptibility, a comprehensive electronic questionnaire was designed for both children and their parents or guardians.

For the children, basic characteristics including age, sex, region, birth weight, ABO blood type, delivery mode, breastfeeding duration, daily sleep duration, daily sleep initiation time, secondhand smoke exposure, vitamin D supplement duration, probiotics supplementary, and average daily screen time were recorded. Children’s body weight (to the nearest 0.1 kg) and height (to the nearest 0.1 cm) were measured by trained health physicians. The hyperactivity and inattention domains of the children were assessed using the Conner’s Abbreviated Symptom Questionnaire (C-ASQ).

For the parents or guardians, sex and self-reported data, including age, weight, height, education, family income, maternal prepregnancy weight, gestational weight gain (GWG), maternal pregnancy smoking exposure, and parental self-rating for their patience with their children, which ranged from 0 to 10 scores, were recorded.

### Sample Size Estimation

According to the method proposed by Riley et al. (2020), in this present study, the estimation of sample size is based on the conditions that overall proportion of children with ADHD susceptibility is 0.05, the number of candidate predictor parameters is 24, and the recommended max Cox-Snell R squared statistic (*R*^2^_cs_) value is 0.33. Assuming that the new model can explain 15% of the variability, the anticipated *R*^2^_cs_ value should be 0.15 × 0.33, equivalent to 0.05. On the basis of the above parameters, the sample size is estimated to be 4,199, corresponding to 210 events and an events per candidate predictor parameter of 8.75.

In the present study, the aforementioned self-designed questionnaires were sent to the parents or guardians of 10,441 children, who read and signed an informed consent form prior to participation, and 98% of them (*n* = 10,230) returned the questionnaires within the scheduled time. After excluding 1,810 questionnaires that had no C-ASQ, 8,420 valid questionnaires were obtained within the specified time frame, the number exceeding the estimated sample size 4,199.

### Quality Control

After training by medical and epidemiological researchers in advance, the kindergarten teachers were responsible for contacting parents or guardians to introduce the questionnaires in detail. All missing/uncertain records were checked by the epidemiological researchers who were responsible for this study, and the kindergarten teachers were contacted to reconfirm the records. Data were exported from electronic questionnaires to a Microsoft Office Excel™ spreadsheet, and were cross-checked by trained staff. After excluding situations with no comprehensive information, incorrect filling and inability to be reconfirmed, mothers who suffered from prenatal and perinatal diseases, and children with a history of disease, including chronic kidney disease, hypothyroidism, congenital heart disease, chronic respiratory diseases, and inherited metabolic diseases, were also eliminated. Finally, 7,938 of the completed questionnaires were deemed eligible for analysis.

### Conner’s Abbreviated Symptom Questionnaire-Defined Attention Deficit Hyperactivity Disorder Susceptibility

The Conner’s Abbreviated Symptom Questionnaire (C-ASQ), a global measure of psychopathology, is not a specific indicator of ADHD diagnosis (Conners, 1989), yet it has a high diagnostic ability in distinguishing children with and without ADHD (Chang et al., 2016). The information obtained from C-ASQ can facilitate the process of determining the requirements for a more comprehensive evaluation. The C-ASQ contains 10 identical items for parent and teacher rating scales, with each item being rated on a 4-point scale of frequency from never or rarely (0) to very often (3). The continuous measure of total ADHD symptoms is calculated as the sum of the score of each item. In this study, we defined children with a point >10 as the propensity of hyperactivity, called C-ASQ-defined ADHD susceptibility (Xiangdong, 1993).

Daily sleep duration was calculated as the sum of both break time during the day and sleep time at night, combining the time on working days × 5 and the corresponding time on weekends × 2 divided by 7. Daily sleep initiation time was grouped into before and after 23:00. Sleep mode was defined according to both sleep duration and daily sleep initiation time and was divided into 4 stages. Sleep mode was encoded as the number “4” if the sleep duration was less than 10 h per day and fall asleep time was after 23:00, as the number “3” if sleep duration was more than 10 h per day and the daily sleep initiation time was after 23:00, as the number “2” if sleep duration was less than 10 h and the daily sleep initiation time was before 23:00, and as the number “1” if the sleep duration was more than 10 h and the daily sleep initiation time was before 23:00. The children’s average daily screen time was calculated as for the daily sleep duration. Secondhand smoking exposure meant that guardians living with the child were long-time smokers, and it was divided into 1–5 cigarettes per day, 5–10 cigarettes per day, and > 10 cigarettes per day. Vitamin D supplement duration was classified into ≤ 3 months, 3–6 months, 6–12 months, and > 12 months.

GWG was calculated from antepartum weight minus the prepregnancy weight, and it included three classes: adequate GWG (weight gain of 12.5–18.0 kg in underweight mothers, 11.5–16.0 kg in normal-weight mothers, 7.0–11.5 kg in overweight mothers, and 5.0–9.0 kg in obese mothers), inadequate GWG (less than the lower limits of adequate levels), and excessive GWG (greater than the upper limits of adequate levels). All reference criteria were based on the recommendations of the Institute of Medicine (2009) (Council, 2010).

Family income (RMB per year) was classified into ≤ 100,000, 100,000–300,000, 300,000–600,000, 600,000–900,000, and >1,000,000. Parent education was defined as a high school degree or below, college degree, master’s degree, and doctor’s degree and above. Smoking exposure during maternal pregnancy included secondhand smoke exposure on the mother and the mother smoking. Scores of parent self-rating about their patience with their children ranged from 0 to 10. A score of 0 indicates no patience. Stages of parent self-rating for the patience were classified into four stages based on the parent self-rating scores: stage 1 (scores = 0–3); stage 2 (scores = 4–6); stage 3 (scores = 7–9); and stage 4 (score = 10).

### Statistical Analyses

The STATA software (version 14.0, Stata Corp., TX) and the R programming environment (version 4.1.0) were used for statistical analyses. All continuous variables were tested for normality and were log-transformed as appropriate, and the mean (SD) was calculated from the raw/non-log-transformed data. Skewed continuous variables were expressed as median (interquartile range). The intraclass correlation coefficient (ICC) was calculated to assess the possibility of non-random measurement error for factors under study collected from 30 public kindergartens. The ICC ranges from 0 to 1, and an ICC of 0 indicates that the variance in factors under study is not due to variation between the kindergartens.

Univariate analyses, specifically the *t*-test or rank-sum test for continuous variables and the chi-square for discrete variables, respectively, were performed for baseline characteristics’ comparison. All statistical tests were two-sided with 0.05 significance levels. Dose–response relationships between children’s breastfeeding duration and C-ASQ-defined ADHD susceptibility were investigated using restricted cubic spline models.

To identify the contribution of all possible factors, the least absolute shrinkage and selection operator (LASSO) model (Tibshirani, 1996), a regression-based methodology permitting a large number of covariates, was used to reduce the likelihood of overfitting and to remove unnecessary/uninfluential covariates. We utilized the “glmnet” package (version 2.0–16) to fit the logistic LASSO regression and used 10-fold cross-validation to select the penalty term, λ. Then, we identified statistically significant factors selected from the logistic LASSO model that were further adjusted for the age of the children, their sex, parents’ age and education, and family income. Effect size estimates were denoted as odds ratios (ORs) and 95% CIs.

We searched for predictors of ADHD symptoms that were repeatedly reported in studies or systematic reviews, which included family income, screen time, low birth weight, probiotic supplementation, and parental education (Banerjee et al., 2007; Russell et al., 2016; Tamana et al., 2019; Hartel et al., 2020). These factors were included in the basic model, and some baseline characteristics, including sex, age, region, BMI, parents’ age at birth, parents’ BMI, delivery mode, GWG, children’s probiotic supplementation, and vitamin D supplement duration, were also included in the basic model. The Akaike information criterion (AIC), Bayesian information criterion (BIC), the −2 log likelihood ratio test, and Hosmer–Lemeshow goodness-of-fit test were used to appraise how closely the prediction probability was obtained by adding significant predictors selected by logistic LASSO regression. Integrated discrimination improvement (IDI) and the area under the receiver operating characteristic (AUROC) curve were used to determine whether the addition of significant predictors can differentiate preschool-aged children with C-ASQ-defined ADHD susceptibility.

The R programming environment (version 4.1.0) “rms” package was used to create a prediction nomogram model for the early identification of C-ASQ-defined ADHD susceptibility. The concordance index or C-index, defined as the AUROC curve, was used to quantify the model predictive accuracy.

Sample size was estimated using the “pmsampsize” package in the STATA software (version 14.0, Stata Corp., TX), which was developed by Riley et al. (2020). Meanwhile, study power was estimated using the PS-Power Simple Size software (version 3.1.2).

## Results

### Baseline Characteristics

The flow diagram to selection of study children could be found in Supplementary Figure 1. The distributions of demographic, prenatal, and perinatal factors and environmental-related factor data from 7,938 children are shown in Table 1.

**TABLE 1:** Baseline characteristics of study participants in this study.

Characteristics	C-ASQ-defined ADHD susceptibility		P
	No (*n* = 6,366)	Yes (*n* = 1,573)	
***For children***			
Age, years			0.002
3–5 years	4,008 (60.3%)	1,057 (67.2%)	
5–7 years	2,357 (37.0%)	516 (32.8%)	
Boys	3,157 (49.6%)	949 (60.3%)	<0.001
Region			<0.001
Beijing	3,563 (56.0%)	1,082 (68.9%)	
Hebei	2,796 (44.0%)	489 (31.1%)	
ABO blood types			0.770
A	977 (28.0%)	264 (26.8%)	
B	1,172 (33.5%)	347 (35.2%)	
O	1,041 (29.8%)	288 (29.2%)	
AB	303 (8.7%)	87 (8.8%)	
Birth weight (kg)	3.3 (3.0, 3.6)	3.3 (3.0, 3.6)	0.529
Delivery mode			0.008
Natural delivery	3,229 (50.7%)	857 (54.5%)	
Artificial midwifery	3,137 (49.3%)	716 (45.5%)	
BMI (kg/m^2^)	15.5 (14.5, 16.8)	15.5 (14.5, 16.7)	0.148
Breastfeeding duration (months)	12.0 (7.0, 18.0)	12.0 (8.0, 18.0)	<0.001
Fall asleep time (hours)			0.012
Before 23:00 pm	6,241 (98.0%)	1,526 (97.0%)	
After 23:00 pm	125 (2.0%)	47 (3.0%)	
Sleep duration	10.0 (9.0, 10.3)	10.0 (9.0, 10.6)	<0.001
Secondhand smoke exposure			<0.001
No	3,537 (55.6%)	757 (48.1%)	
1–5 cigarettes per day	1,907 (29.9%)	493 (31.3%)	
5–10 cigarettes per day	553 (8.7%)	195 (12.4%)	
> 10 cigarettes per day	369 (5.8%)	128 (8.1%)	
Vitamin D supplement duration			0.001
≤ 3 months	1,450 (26.8%)	317 (22.4%)	
3–6 months	969 (17.9%)	240 (16.9%)	
6–12 months	1,061 (19.6%)	304 (21.5%)	
> 12 months	1,935 (35.7%)	556 (39.2%)	
Probiotics supplemented			<0.001
Yes	2,079 (32.7%)	395 (25.1%)	
No	4,287 (67.3%)	1,178 (74.9%)	
Screen time (h/per day)	1.0 (0.6, 1.6)	1.0 (0.9, 2.0)	<0.001
***For parents or guardians***			
Family income (RMB per year)			<0.001
≤100,000	2,636 (41.4%)	546 (34.7%)	
100,000–300,000	2,382 (37.4%)	678 (43.1%)	
300,000–600,000	995 (15.6%)	261 (16.6%)	
600,000–900,000	229 (3.6%)	61 (3.9%)	
>1,000,000	124 (2.0%)	27 (1.7%)	
Maternal education			<0.001
High school degree or below	2,600 (40.8%)	474 (30.1%)	
College degree	3,220 (50.6%)	971 (61.7%)	
Master degree	485 (7.6%)	113 (7.2%)	
Doctor degree and above	61 (1.0%)	15 (1.0%)	
Paternal education			<0.001
High school degree or below	2,845 (44.7%)	574 (36.5%)	
College degree	2,933 (46.1%)	847 (53.8%)	
Master degree	492 (7.7%)	126 (8.0%)	
Doctor degree and above	96 (1.5%)	26 (1.7%)	
Maternal BMI (kg/m^2^)	22.3 (20.3, 24.8)	22.5 (20.5, 24.8)	0.084
Paternal BMI (kg/m^2^)	25.4 (22.9, 28.4)	25.3 (22.9, 27.8)	0.045
Maternal age while children birth	28.5 (26.3, 31.2)	29.0 (26.8, 32.1)	<0.001
Paternal age while children birth	29.4 (27.1, 32.5)	30.0 (27.5, 33.5)	<0.001
Gestational weight gain (kg/m^2^)			0.011
Inadequate	154 (24.3%)	380 (24.2%)	
Adequate	2,159 (33.9%)	477 (30.3%)	
Excessive	2,661 (41.8%)	716 (45.5%)	
Maternal pregnancy smoking			<0.001
Yes	3,766 (59.2%)	1,051 (66.8%)	
No	2,600 (40.8%)	522 (33.2%)	
Parental self-rating for patience			<0.001
1–3 points	254 (4%)	131 (8.3%)	
4–6 points	1,775 (27.9%)	653 (41.5%)	
7–9 points	3,526 (55.4%)	722 (45.9%)	
10 points	811 (12.7%)	67 (4.3%)	

*Abbreviations: BMI, body mass index. Data are expressed as median (interquartile range) or count (percent). P value was calculated by the rank-sum test or the χ^2^ test, where appropriate.*

In addition, as study children were from 30 public kindergartens, the potential bias arising from different kindergartens was evaluated using the ICC. For 24 factors under study, ICC values ranged from 0.001 to 0.02, indicating a low likelihood of potential bias.

### Identification of Contributing Predictors

**FIGURE 1:**
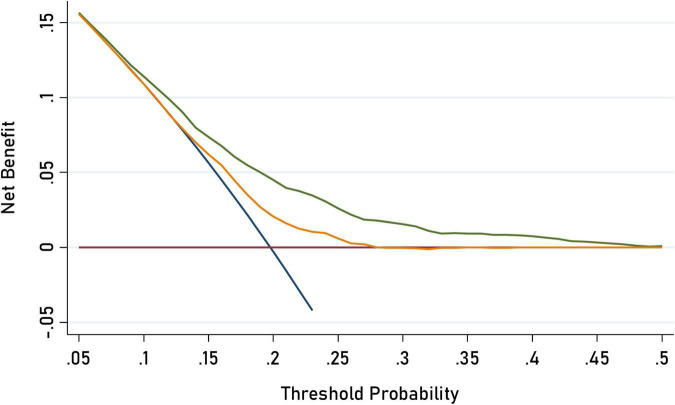
Decision curve analysis for C-ASQ-defined ADHD susceptibility by adding significant factors to the basic model. The orange solid line corresponds to the basic model that includes sex, age, region, BMI (body mass index), family income, maternal education, and parental education, parent’s age while children birth, parent’s BMI, delivery mode gestational weight gain, probiotics supplemented, vitamin D supplement duration, screen time. The green solid line corresponds to the full model that includes both factors in the basic model and the five newly- identified significant factors including children’s second hand smoking exposure, and parental self-rating patience. Larger area between the two lines represents better accuracy of the full prediction model.

The λ values for the logistic LASSO regression ranged from 0.000388 to 0.074940, and the selected λ value was 0.006509 in our study (Supplementary Figure 2). Originally, there were 10 variables in the LASSO regression model, including age, sex, maternal age at birth, paternal age at birth, secondhand smoking exposure, delivery mode, breastfeeding duration, sleep mode, maternal pregnancy smoking exposure, and parental self-rating for patience. These variables were further adjusted and corrected. Finally, five factors, namely, children’s secondhand smoking exposure (OR: 1.24; 95% CI: 1.16–1.32; *p* < 0.001), breastfeeding duration (OR: 0.98; 95% CI: 0.98–0.99; *p* < 0.001), sleep mode (OR: 1.22; 95% CI: 1.17–1.28; *p* < 0.001), maternal pregnancy smoking exposure (OR: 1.28; 95% CI: 1.13–1.46; *p* < 0.001), and parental self-rating for patience (OR: 0.51; 95% CI: 0.47–0.56; *p* < 0.001), were independently identified by the multiple adjustment to be significantly associated with C-ASQ-defined ADHD susceptibility (Figure 1 and Table 2).

**TABLE 2:** Risk prediction of five significant factors for C-ASQ-defined ADHD susceptibility.

Variables	C-ASQ-defined ADHD susceptibility		
	OR	95% CI	P
**Unadjusted**			
Secondhand smoke exposure	1.21	1.14–1.28	<0.001
Parental self-rating for patience	0.56	0.52–0.60	<0.001
Breastfeeding duration	0.98	0.98–0.99	<0.001
Maternal pregnancy smoking	1.39	1.24–1.56	<0.001
Sleep mode	1.23	1.17–1.28	<0.001
Partial adjustment*			
Secondhand smoke exposure	1.23	1.16–1.31	<0.001
Parental self-rating for patience	0.52	0.48–0.56	<0.001
Breastfeeding duration	0.98	0.98–0.99	<0.001
Maternal pregnancy smoking	1.31	1.15–1.48	<0.001
Sleep mode	1.22	1.17–1.28	<0.001
Multiple adjustment**			
Secondhand smoke exposure	1.24	1.16–1.32	<0.001
Parental self-rating for patience	0.51	0.47–0.56	<0.001
Breastfeeding duration	0.98	0.98–0.99	<0.001
Maternal pregnancy smoking	1.28	1.13–1.46	<0.001
Sleep mode	1.22	1.17–1.28	<0.001

Abbreviations: OR, odds ratio; 95% CI, 95% confidence interval. *Partial adjustment contained sex, age, region, BMI (body mass index), family income, maternal education, and paternal education. **Multiple adjustment additionally included ABO blood types of children, children’s birth weight, delivery mode, probiotics supplemented, Vitamin D supplement duration, parents’ age while children birth, parents’ BMI, gestational weight gain.

On the basis of the above effect-size estimates, the minimal power to detect the significant association of the above five factors with ADHD risk was over 81.3%.

### Dose–Response Analysis

**FIGURE 2:**
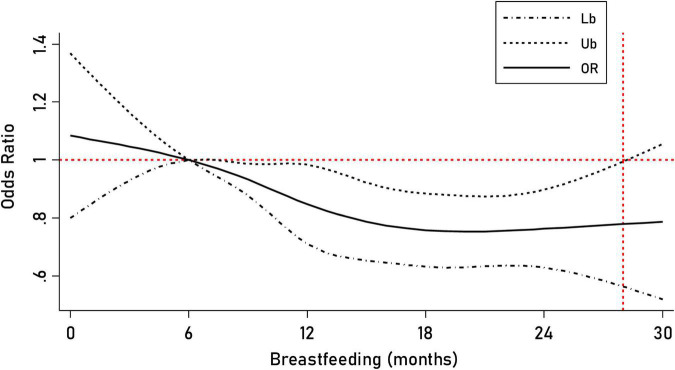
Prediction of the breastfeeding duration for C-ASQ-defined ADHD susceptibility. The solid line represents the odd ratio (OR) for C-ASQ-defined ADHD susceptibility as the breastfeeding duration increases, and the black dashed line represents the corresponding 95% confident interval of the odd ratio.

We found a dose–response relationship between childhood breastfeeding duration and C-ASQ-defined ADHD susceptibility (Figure 2). Six months of breastfeeding duration was such a dramatic threshold; children seemed more likely to suffer from ADHD symptoms if the breastfeeding duration was less than 6 months.

In terms of sleep model, we found that in children that fell asleep before 23:00 and had a sleep duration of more than 8 h but less than 10 h, their odds ratio for ADHD susceptibility was 1.27 (95% CI: 1.13–1.44, *p* < 0.001) compared to those who fell asleep before 23:00 combined with sleep duration of more than 10 h. Likewise, serious trends were observed for a shorter sleep duration and a later sleep time [falling asleep time < 23:00 and sleep duration ≤ 8 h (OR: 1.54; 95% CI: 1.25–1.89; *p* < 0.001); falling asleep time ≥ 23:00 and sleep duration > 10 h (OR: 0.94; 95% CI: 0.52–1.72; *p* = 0.85); fall asleep time ≥ 23:00 and 8 h < sleep duration < 10 h (OR: 2.19; 95% CI: 1.32–3.62; *p* = 0.002); fall asleep time ≥ 23:00 and sleep duration ≤ 8 h (OR: 3.59; 95% CI: 1.62–7.94; *p* = 0.002)] (Supplementary Table 1). These results were adjusted by other variables investigated in this survey.

Meanwhile, the risk of having ADHD-related symptoms was proportional to cigarette exposure of the children. Compared to never exposure, the risk ratio of children’s exposure to 1–5 cigarettes per day was 1.20, with a 95% CI ranging from 0.98 to 1.46 (*p* = 0.08), while for those exposed to 5–10 and > 10 cigarettes per day, the risk ratio increased (OR: 1.48; 95% CI: 1.12–1.94; *p* = 0.005 and OR: 1.45; 95% CI: 1.04–2.02; *p* = 0.03, respectively).

For parental self-rating for patience, we concluded that children had ADHD-related symptoms when decreasing the stages of the self-rating. Compared with stage 1, the odds ratio was 0.62 (95% CI: 0.42–0.92; *p* = 0.02) in stage 2, 0.35 (95% CI: 0.24–0.52; *p* < 0.001) in stage 3, and 0.14 (95% CI: 0.08–0.24; *p* < 0.001) in stage 4.

### Prediction Accuracy Assessment

A basic model and a full model were constructed to synthetically assess the prediction accuracy of the five significant factors. The full model included all variables investigated in this survey, and the basic model included all variables except the five factors. As shown in Table 3, the prediction accuracy gained by adding five significant factors associated with C-ASQ-defined ADHD susceptibility in preschool children was separately assessed by calibration and discrimination statistics. Compared with the basic model, the prediction accuracy was significantly improved in the full model and the difference was significant for the prediction performance in parallel.

**TABLE 3:** Prediction accuracy gained by adding five significant factors associated with C-ASQ-defined ADHD susceptibility in pre-school children.

Statistics	C-ASQ-defined ADHD susceptibility	
	Basic model	Full model
**Calibration**		
AIC	7480.1	7131.0
BIC	7556.4	7242.0
LR test (χ^2^)	354.02	
LR test (*P*-value)	<0.0001	
**Discrimination**		
NRI	<0.0001	
IDI	<0.0001	
AUROC curves (*P*-value)	<0.0001	

*Abbreviations: AIC, Akaike information criterion; BIC, Bayesian information criterion; LR, likelihood ratio; NRI, net reclassification improvement; IDI, integrated discrimination improvement; ROC, area under the receiver operating characteristic. Basic model included sex, age, region, BMI (body mass index), family income, maternal education, and paternal education, parents’ age while children birth, parents’ BMI, delivery mode, gestational weight gain, probiotics supplemented, vitamin D supplement duration, screen time.*

### Prediction Nomogram Model

**FIGURE 3:**
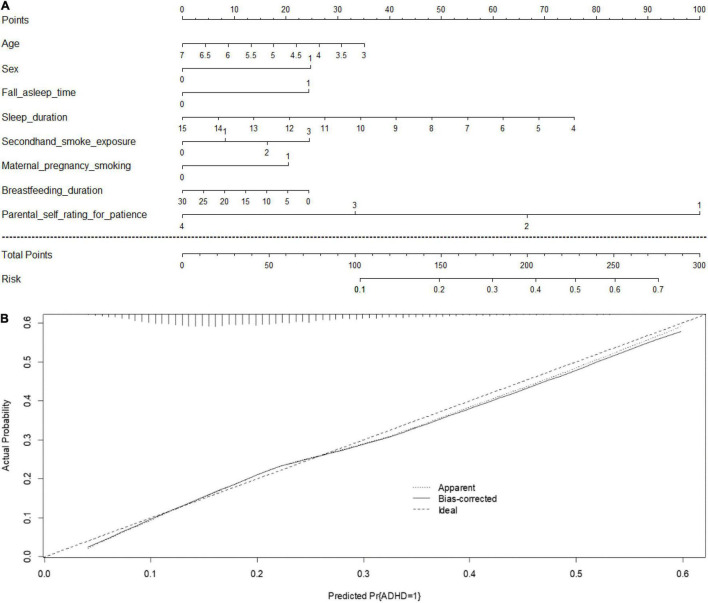
**(A)** Prediction nomogram for C-ASQ-defined ADHD susceptibility. An individual’s value is located on each variable axis, and a line is drawn upward to determine the number of points received for each variable value. The sum of these numbers is located on the “Total points” axis, and a line is drawn downward to the “Risk” axis to determine the likelihood of C-ASQ-defined ADHD susceptibility. **(B)** Calculation curves for C-ASQ-defined ADHD susceptibility. Nomogram- predicted probability of C-ASQ defined ADHD susceptibility plotted on the x- axis; actual probability of C-ASQ defined ADHD- susceptibility on the y- axis. The plots depict model performance in terms of agreement between predicted and actual probability of C-ASQ defined ADHD susceptibility. Prediction is represented by the 45 degree line.

A prediction nomogram model is necessary for extensive preliminary application of the C-ASQ-defined ADHD susceptibility in preschool children. Combining the five significant factors and the children’s age and sex, we created a nomogram model (Figure 3A), with a concordance index (C-index) of 71% (*p* < 0.05), and good predictive accuracy (Figure 3B).

An example to enhance its practical application is as follows: assume a boy (25 points) aged 5 years old (18 points), who falls asleep after 23:00 each day (24 points), with a sleep duration of 10 h (34 points), exposed to secondhand smoking 5–10 cigarettes per day (17 points), with a breastfeeding duration of 5 months (20 points), maternal pregnancy smoking exposed (20 points), and stage 4 of parental self-rating for practice being 1–3 (100 points). Since the total point is 258, the probability of developing ADHD-related symptoms is estimated to be approximately 64%.

## Discussion

Using a cross-sectional representative survey, annexed with robust statistical techniques, we aimed to identify and characterize prenatal and perinatal factors and environmental-related factors for C-ASQ-defined ADHD susceptibility among 7,938 preschool-aged children. The key point of our study is that five significant factors, namely, children’s secondhand smoking exposure, breastfeeding duration, sleep mode, maternal pregnancy smoking exposure, and parental self-rating for patients, were independently associated with ADHD susceptibility assessed by Conner’s ASQ. To our knowledge, this study is the first investigation about screening for ADHD-related symptoms in preschool-aged children in China.

In China, great efforts are being made to improve children’s mental health, and the prevalence estimates of ADHD in mainland China, Hong Kong, and Taiwan were 6.5, 6.4, and 4.2%, respectively (Liu et al., 2018), which were derived from 67 studies covering 642,266 Chinese children and adolescents. However, little is known about the prevalence of ADHD in children aged 3–7 years. Due to the rapid physical, emotional, behavior, and cognitive development that young children experience, their emotional and behavior problems are often considered transient problems rather than mental disorders (Angold and Egger, 2004). However, many studies have proposed that preschoolers’ temperaments and patterns of temperament traits could be linked to increased risks for later psychiatric disorders (Hirshfeld-Becker et al., 2003), and the rates of common childhood psychiatric disorders in preschoolers are similar to those seen in later childhood (Egger and Angold, 2006). Screening for ADHD susceptibility as well as searching for influential factors is necessary for the early detection, prevention, and intervention of childhood ADHD, as studies have demonstrated that children who did not undergo screening had a higher incidence of cognitive and behavior problems than those who did (Burakevych et al., 2016).

A growing number of studies have examined the risk factors associated with ADHD-related symptoms, yet the results are often not reproducible. For example, some studies have shown a significant association between maternal exposure to smoking and ADHD (Sourander et al., 2019), whereas others have failed to support this claim (Gustavson et al., 2017), with different sample sizes and regions, and possible incomplete adjustment of measured or unmeasured covariates.

The present study accounted for this aspect and undertook logistic LASSO regression and a hierarchical degree of adjustment for possible covariates, and added to existing evidence that co-exposure to prenatal and perinatal factors and environmental related factors significantly influenced ADHD-related outcomes. First, we found that smoking exposure was a severe risk factor, including maternal pregnancy exposure to smoking and children directly exposed to secondhand smoking. Many studies, including umbrella reviews, have proposed a relationship between nicotine exposure during pregnancy and offspring ADHD and have found a dose–response relationship (Thakur et al., 2013; Sourander et al., 2019; Kim et al., 2020). Meanwhile, a meta-analysis illustrated that postnatal exposure to secondhand smoking increased the risk of ADHD in children (OR: 1.60; 95% CI: 1.37–1.87) (Huang et al., 2021). These findings are highly consistent with our results.

Second, in terms of sleep mode, many studies have concluded that a short sleep duration is significantly linked to ADHD compared with an average sleep duration (Lee et al., 2019), especially in early childhood (Tso et al., 2019). Cremone-Caira et al. (2020) suggested that a sleep extension intervention improves inhibitory control in children with ADHD. Some studies have also illustrated that children’s sleep onset latency, bedtime resistance, and sleep onset difficulties are significantly higher in children with ADHD (Cortese et al., 2009). In our study, we sought an interactive relationship between sleep duration and sleep initiation time, and found that the risk for ADHD susceptibility rose dramatically with a short sleep duration and a later bedtime.

Protective factors were also prominent in our study. A breastfeeding duration over 6 months is associated with lower conduct disorders. In early 1998, the World Health Organization (WHO) Expert Consultation recommended that infants be exclusively breastfed during the first 6 months of life (World Health Organization, 1998). Prolonged breastfeeding of infants may promote their mental health later in childhood, particularly by reducing the risk for inattention/hyperactivity and conduct disorders (Hartel et al., 2020). Breastfeeding could foster immune–microbiome interplay and promote mother–child interactions as well as improve long-term neurobehavior outcomes (Agostoni et al., 2017; Boucher et al., 2017; Tseng et al., 2019). Similarly, parents’ patience is a significant factor for childhood development, especially for mental health. In our study, a higher parental self-rating for patience was associated with a lower risk of suffering from ADHD susceptibility. Multiple studies have shown that ADHD is linked to parent–child conflicts (Pelham and Fabiano, 2008; Williamson and Johnston, 2016), and ineffective, inconsistent, negligent parenting was found to exacerbate ADHD symptoms and be predictive of later disruptive behavior disorders (Ullsperger et al., 2016).

Finally, a risk prediction nomogram model for C-ASQ-defined ADHD susceptibility was created to enhance the practical applications of our findings. This model has excellent forecast precision and will be useful for conducting preliminary screening. However, several limitations of our study merit special consideration. First, since it is a cross-sectional study, it precludes further comments on the cause–effect relationship. We could not ascertain whether short sleep duration was a risk factor or a consequence of ADHD in this study, but we argued from an epidemiological perspective that sleep duration has an effect on preschool-aged children’s ADHD susceptibility. Second, ADHD is a clinical diagnosis requiring a detailed evaluation of current and previous symptoms and a functional impairment, and full family, gestational, and developmental history should be taken (Barkley, 2002). The key point of the present study focused on preliminary screening, and further diagnosis requires repeated evaluations. Third, objectively monitored variables such as sleep duration were self-reported by parents, and hence, recall bias could not be ruled out. Finally, some important items such as a family history of ADHD and the number of siblings were not surveyed in this study. In subsequent studies, we will pay more attention to hereditary factors.

In summary, *via* an analysis of survey data from 7,938 preschool-aged children and their parents or guardians, we identified five factors independently and consistently associated with children’s C-ASQ-defined ADHD susceptibility, and a risk prediction nomogram model was established.

## Conclusion

In general, five factors, namely, children’s secondhand smoking exposure, breastfeeding duration, sleep mode, maternal pregnancy smoking exposure, and parental patience self-rating, were independently associated with ADHD susceptibility. These findings will help with developing intervention strategies for preventing ADHD susceptibility by promoting parental patience and encouraging a longer sleep duration and an earlier bedtime, a sufficient breastfeeding duration, and no secondhand smoking exposure.

## Data Availability Statement

The raw data supporting the conclusions of this article will be made available by the authors, without undue reservation.

## Ethics Statement

The studies involving human participants were reviewed and approved by the Ethics Committee of China-Japan Friendship Hospital. Written informed consent to participate in this study was provided by the participants’ legal guardian/next of kin.

## Author Contributions

KW, ZZ, and WN conceived and designed the experiments. XD and MY performed the experiments. XD and WN analyzed the data and wrote the manuscript. XD, MY, and BZ contributed materials and analysis tools. All authors read and approved the final manuscript prior to submission.

## Conflict of Interest

The authors declare that the research was conducted in the absence of any commercial or financial relationships that could be construed as a potential conflict of interest.

## Publisher’s Note

All claims expressed in this article are solely those of the authors and do not necessarily represent those of their affiliated organizations, or those of the publisher, the editors and the reviewers. Any product that may be evaluated in this article, or claim that may be made by its manufacturer, is not guaranteed or endorsed by the publisher.
