# Supplementary material for: Identification and Characterization of Influential Factors in Susceptibility to Attention Deficit Hyperactivity Disorder Among Preschool-Aged Children
Source: Front Neurosci. 2022 Jan 31;15:709374. doi: 10.3389/fnins.2021.709374 (PMC8841729; doi:10.3389/fnins.2021.709374)
Supplement: Supplementary file 1 [file Data_Sheet_1.docx]

**Supplementary Table 1.** The interaction of secondhand smoke exposure, maternal pregnancy smoking, breastfeeding, sleep mode, patience in predicting the risk of C-ASQ-defined ADHD susceptibility in preschool-aged children.

| **Interaction items** | **C-ASQ-defined ADHD susceptibility** | | |
| --- | --- | --- | --- |
|  | **OR** | **95% CI** | **P** |
| ***Secondhand smoke exposure (Ssmoke) & Maternal pregnancy smoking (Msmoke)*** | | | |
| Without Ssmoke / Without Msomke | Ref. | | |
| With Ssmoke / Without Msomke | 1.42 | 1.18 to 1.72 | <0.001 |
| Without Ssmoke / With Msomke | 1.45 | 1.23 to 1.71 | <0.001 |
| With Ssmoke / With Msomke | 1.88 | 1.60 to 2.22 | <0.001 |
| ***Fall asleep time (F time) & Sleep duration (S duration)*** | | | |
| F time < 23:00 pm / S duration ≥10h | Ref. | | |
| F time < 23:00 pm / 8h< S duration <10h | 1.27 | 1.13 to 1.44 | <0.001 |
| F time < 23:00 pm / S duration ≤8h | 1.54 | 1.25 to 1.89 | <0.001 |
| F time ≥ 23:00 pm / 8h< S duration <10h | 2.19 | 1.32 to 3.62 | 0.002 |
| F time ≥ 23:00 pm / S duration ≤8h | 3.59 | 1.62 to 7.94 | 0.002 |
| ***Breastfeeding duration (B duration) & Maternal pregnancy smoking (Msmoke)*** | | | |
| B duration <6 months / With Msomke | Ref. | | |
| B duration <6 months / Without Msomke | 1.16 | 0.86 to 1.55 | 0.33 |
| B duration ≥6 months / With Msomke | 0.96 | 0.79 to 1.17 | 0.70 |
| B duration ≥6 months / Without Msomke | 0.64 | 0.52 to 0.79 | <0.001 |
| ***Breastfeeding duration (B duration) & Secondhand smoke exposure (Ssmoke)*** | | | |
| B duration <6 months / With Ssmoke | Ref. | | |
| B duration <6 months / Without Ssmoke | 0.76 | 0.57 to 1.00 | 0.05 |
| B duration ≥6 months / With Ssomke | 0.80 | 0.65 to 0.99 | 0.04 |
| B duration ≥6 months / Without Ssomke | 0.59 | 0.48 to 0.73 | <0.001 |
| ***Fall asleep time (F time) & Parental self-rating for patience (P patience)*** | | | |
| F time <23:00 pm / P patience ≥7 points | Ref. | | |
| F time <23:00 pm / P patience <7 points | 2.10 | 1.87 to 2.35 | <0.001 |
| F time ≥23:00 pm / P patience ≥7 points | 1.22 | 0.74 to 2.12 | 0.44 |
| F time ≥23:00 pm / P patience <7 points | 3.96 | 2.42 to 6.48 | <0.001 |

**Abbreviations:** OR, odds ratio; 95% CI, 95% confidence interval; Ref., reference group. All effect-size estimates were adjusted for age, sex, and region.

**Supplementary Table 2.** The dose-response analysis of cigarette exposure of the children, sleep mode, patience in predicting the risk of C-ASQ-defined ADHD susceptibility in preschool-aged children.

| **Dose-response analysis** | **C-ASQ-defined ADHD susceptibility** | | |
| --- | --- | --- | --- |
|  | **OR** | **95% CI** | **P** |
| ***Cigarette exposure of the children*** | | | |
| Without smoke | Ref. | | |
| 1–5 cigarettes per day | 1.20 | 0.98 to 1.46 | 0.080 |
| 5-10 cigarettes per day | 1.48 | 1.12 to 1.94 | 0.005 |
| >10 cigarettes per day | 1.45 | 1.60 to 2.22 | <0.001 |
| ***Sleep Mode: Fall asleep time (F time) & Sleep duration (S duration)*** | | | |
| F time < 23:00 pm / S duration ≥10h | Ref. | | |
| F time < 23:00 pm / 8h< S duration <10h | 1.27 | 1.13 to 1.44 | <0.001 |
| F time < 23:00 pm / S duration ≤8h | 1.54 | 1.25 to 1.89 | <0.001 |
| F time ≥ 23:00 pm / 8h< S duration <10h | 2.19 | 1.32 to 3.62 | 0.002 |
| F time ≥ 23:00 pm / S duration ≤8h | 3.59 | 1.62 to 7.94 | 0.002 |
| ***Parental self-rating for patience*** |  |  |  |
| Stage 1 | Ref. | | |
| Stage 2 | 0.62 | 0.42 to 0.92 | 0.020 |
| Stage 3 | 0.35 | 0.24 to 0.52 | <0.001 |
| Stage 4 | 0.14 | 0.08 to 0.24 | <0.001 |

**Abbreviations:** OR, odds ratio; 95% CI, 95% confidence interval; Ref., reference group. All effect-size estimates were adjusted for sex, age, region, BMI (body mass index), family income, maternal education, and paternal education, parents’ age while children birth, parents’ BMI, delivery mode, gestational weight gain, probiotics supplemented, vitamin D supplement duration, screen time.
