# Supplementary material for: Identification and Characterization of Influential Factors in Susceptibility to Attention Deficit Hyperactivity Disorder Among Preschool-Aged Children
Source: Front Neurosci. 2022 Jan 31;15:709374. doi: 10.3389/fnins.2021.709374 (PMC8841729; doi:10.3389/fnins.2021.709374)

**Supplementary Figure 1.** The flow diagram to selection of study children.

Assessed for eligibility (n=10230)

First around of exclusion (n=2068)

- With no C-ASQ results (n=1810)
- No guardian signed informed consent (n=258)

For further check (n= 8162)

Second around of exclusion (n=223)

- Not meeting inclusion criteria (n=40)
- Missing/uncertain records after reconfirming (n=183)

Analysis (n= 7939)

**Supplementary Figure 2.** Identification of significant risk factors using LASSO regression analysis.


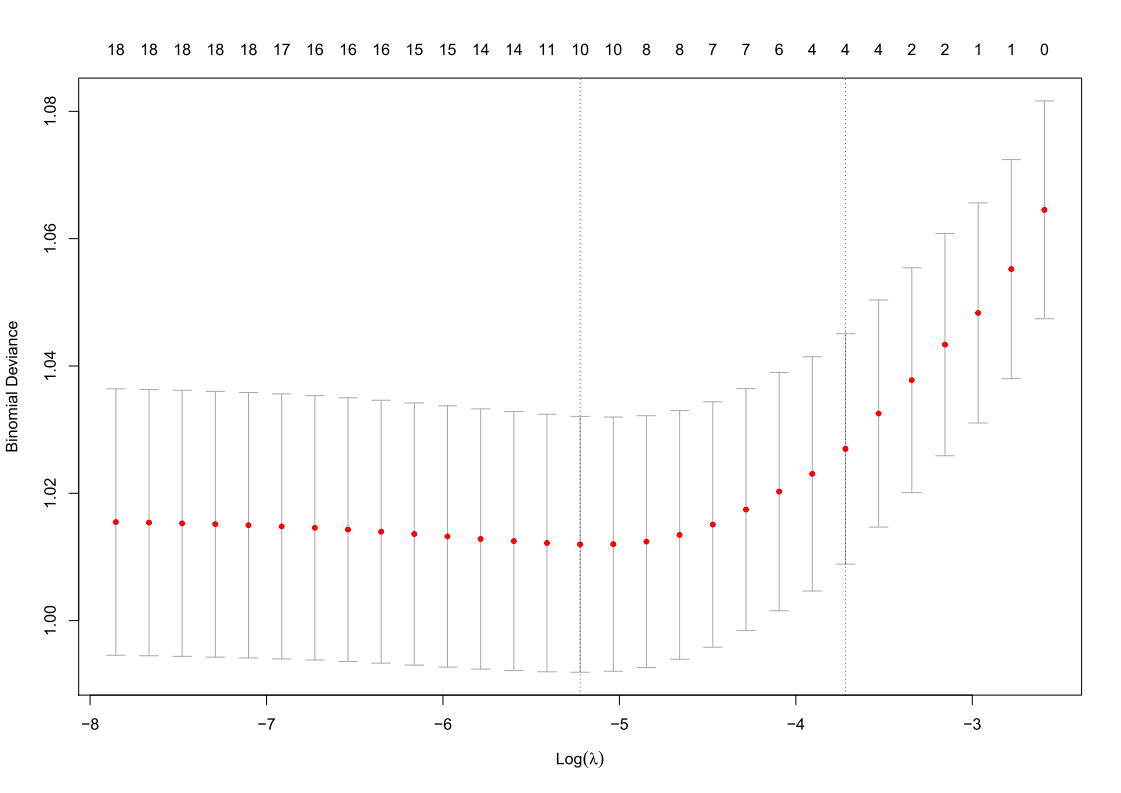

Supplement: Supplementary file 2 [file Data_Sheet_2.docx]
